# Copy Tools in the Electronic Health Record: Perceptions, Implications, and Future Directions

**DOI:** 10.2196/78502

**Published:** 2025-12-19

**Authors:** Sky Corby, Joan S Ash, Rebecca M Jungbauer, Gretchen Scholl, Sarah Florig, Vishnu Mohan, Jeffrey A Gold

**Affiliations:** 1 Division of Pulmonary, Allergy, and Critical Care Medicine Department of Medicine Oregon Health & Science University Portland, OR United States; 2 Division of Informatics, Clinical Epidemiology, and Translational Data Science Department of Medicine Oregon Health & Science University Portland, OR United States; 3 Division of Undergraduate Medical Education Department of Medicine Oregon Health & Science University Portland, OR United States; 4 College of Medicine Nebraska Medical Center Omaha, NE United States; 5 Division of General Internal Medicine and Geriatrics Department of Medicine Oregon Health & Science University Portland, OR United States

**Keywords:** clinical communication, electronic health records, clinical documentation, copy-paste, carry-forward, qualitative research, attitude of health personnel, electronic documentation, human factors

## Abstract

**Background:**

Electronic health records (EHRs) can aid in provider efficiency, but may also lead to unintended consequences, such as documentation burden and increased length of notes. To combat issues related to documentation, copying and pasting (CP) and copying or carrying forward (CF) are tools that have been used to aid in documentation burden. Multiple studies have identified the benefits and challenges of using these tools; however, few studies have identified the unintended consequences of CP and CF, and how the adoption of these tools may affect users.

**Objective:**

The objective was to describe providers’ perceptions and use of copying tools available in the EHR and describe their suggestions for improvement on these copying tools.

**Methods:**

Research team members conducted semistructured interviews with faculty members, advanced practice providers, residents or fellow trainees, and medical students at a single academic health sciences center. The Diffusion of Innovations Theory of Unintended Consequences guided the analysis and interpretation of interview results.

**Results:**

A total of 22 semistructured interviews were conducted in 2023 and analyzed during 2024. The findings showed that respondents use and value these tools for efficiency and communication purposes. The negative unintended consequences include inaccuracies and errors in documentation and increased patient safety risks. Some respondents experience inner angst or moral injury related to using CP/CF, but they feel that they must use them to satisfy organizational requirements surrounding documentation. The respondents suggested that artificial intelligence will likely help improve documentation tools, as would further training around these types of documentation tools.

**Conclusions:**

Some respondents noted feeling both internal and external pressures that influenced when and how they use CP/CF. Respondents noted that they value EHR copying tools for efficiency purposes, but they also understand the risks involved. This tension may lead to moral angst or moral injury. They offered numerous suggestions for lowering the risk, especially by improving the documentation capabilities of the EHR through artificial intelligence. Future research should investigate both technical and educational solutions to relieve the documentation burden and moral angst they are experiencing.

## Introduction

Electronic health records (EHRs) were first introduced as a more efficient mechanism than paper for collecting and storing patient information such as test results, diagnoses, and clinical notes [1]. Although the EHR is effective in many aspects, the adoption of this technology has given rise to unintended consequences. One primary concern, documentation burden, includes increased length of notes (“note bloat”) [2] and time spent documenting [3]. These unintended consequences contribute to another widespread problem: provider burnout [4].

To combat issues related to EHR documentation, vendors have developed and implemented documentation tools that facilitate copying and pasting (CP) text and copying or carrying forward (CF) entire notes [5] into their products that clinical users in health care organizations have embraced. CP allows users to copy elements of a note (eg, free text, test results, and clinical narratives) and paste that information into a new note. CF, available in some EHR systems, is used to copy an entire note, including templates and formats, to bring forward into a new note.

Prior studies suggest CP/CF is used to improve usability and efficiency in documentation [6], to decrease provider burnout [7], and to improve documentation quality, where quality was defined as the accuracy of the information, and timeliness of the information, and the overall readability of the information in the note [8]. However, CP can be a significant contributor to note bloat and error [3,5,9-14]. Some researchers found that 90% or more of inpatient service notes were either copied or templated, increasing length and number of errors in the notes [8,15], while others reported CP/CF-related diagnostic and process errors contributed to the potential for moderate to severe harm [16,17]. Please see Table 1 for a summary comparing intended versus unintended consequences.

**Table 1:** Summary of intended and unintended consequences of using CP^a^/CF^b^ in EHR documentation from previous literature.

Intended consequences of CP/CF	Unintended consequences of CP/CF
Efficiency	Note bloat
Relieve burnout	Increase in errors in documentation
Improve documentation quality	Increase in diagnostic errors

^a^CP: copying and pasting.

^b^CF: copying or carrying forward.

Researchers have typically investigated CP/CF use via surveys [8,10,18,19] and large retrospective analyses of notes [11,12,14,20]. A mixed-methods study assessing the association of CP use and risk severity, as well as perspectives of CP/CF use in progress notes and reported a wide spectrum of the value of CP/CF and progress notes across note authors and consumers was carried out [21]. Other research has found similar variance in perception of value and efficiency by specialty, seniority, and setting [8,10].

**Figure 1:**
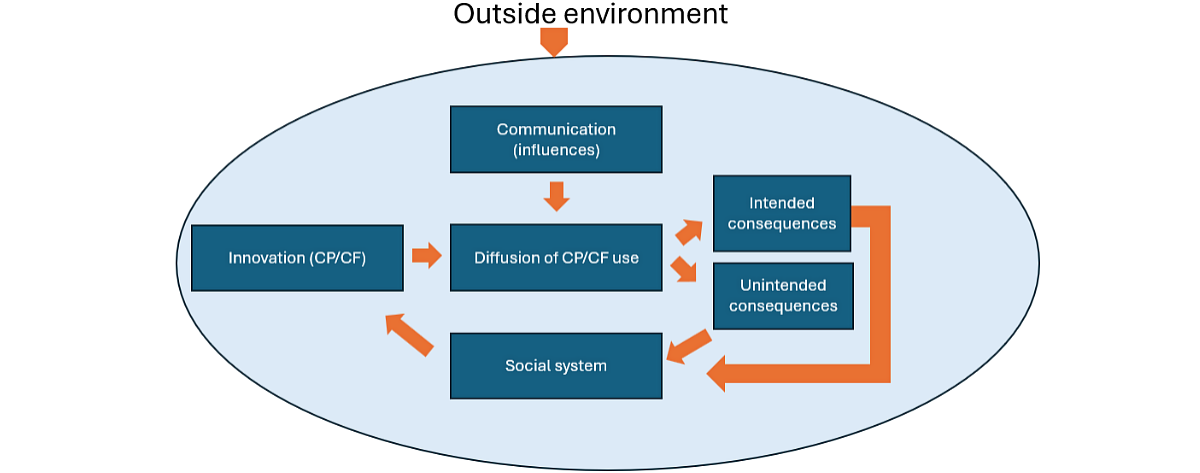
The figure below depicts the modified Diffusion of Innovations Theory of Unintended Consequences, which is a theory showing a circular pattern where an innovation is introduced and adopted. The adoption of innovation then creates the intended and unintended consequences, which are followed by iterative updates based on interactions from the social system and the outside environmental pressures. CF: copying or carrying forward; CP: copying and pasting.

Numerous studies have described the benefits (eg, efficiency) and challenges (eg, note bloat) of these tools. However, evidence considering the context in which these tools are implemented, and the potential to limit or avoid unanticipated and undesirable consequences of implementation, is lacking. The Diffusion of Innovations model posits that adoption of innovations proceeds through stages and is affected by properties of the innovations, adopters, social systems, change agents (individuals pushing for process improvement), and communication relationships [22,23]. While CP/CF may be considered successful innovations in terms of adoption, the unanticipated (and potentially contradictory) consequences of CP/CF use may have dramatic ramifications for users, consumers, and the health systems themselves [22]. This Theory of Unintended Consequences, a corollary to the Diffusion of Innovations Theory, is depicted in Figure 1, showing a cycle in which an innovation (in this case, CP/CF) is introduced and adopted. Adoption creates the intended and unintended consequences described in prior research, followed by iterative updates based on interactions with the social system. Throughout the process, the outside environment (eg, policy or cultural values) exerts pressure against the innovation usage.

However, the benefits and challenges of innovative tools, such as CP/CF, coupled with an understanding of how the unanticipated and unintended consequences of CP/CF adoption affect users, are poorly described in the current literature. We aimed to use qualitative methodologies, guided by the Diffusion of Innovation Theory of Unintended Consequences, to (1) investigate provider perceptions, thoughts, and attitudes about CP/CF in the EHR; (2) identify the consequences (both intended and unintended) of using CP/CF across different specialties, departments, provider roles, and training level in one academic health care system; and (3) better understand users’ experiences with intended and unanticipated consequences of CP/CF by focusing on the futures of CP/CF.

## Methods

### Ethical Considerations

This study was approved by the Institutional Review Board at Oregon Health & Science University (IRB #10027). Informed consent was obtained from respondents invited via email to participate in this study, and verbal consent was given before being recorded during the interview. The data used for this paper were deidentified, and the manuscript does not contain any information that would allow for the identification of respondents. Respondents were informed that study findings would be published, and their quotations would be presented anonymously. Participants were not compensated for participating in the interview.

### Recruitment or Setting of This Study

Respondents for this comprised providers who initially responded to a prior quantitative survey [19] in 2022-2023 regarding use of CP/CF for documentation, and while completing the survey, self-identified to be interviewed for the present study. These providers included faculty, advanced practice providers (APPs), medical students, and residents or fellow trainees at a single academic health institution. For our study, we have defined APPs as health care professionals who have had special training to diagnose, treat, and manage common medical conditions, which include physician’s assistants, certified nurses, nurse practitioners, and licensed social workers. APPs, faculty, medical students, and residents or fellow trainees who all used Epic (Epic Systems); and medical students and residents or fellow trainees on rotation at the Veterans Affairs health care system who also used VistA (Veterans Health Information Systems and Technology Architecture; US Department of Veterans Affairs). Thus, while the initial survey was purposively sampled, the participants identified for this study represented a convenience subsampling of a prior purposively selected cohort. The research team reached out to individuals who volunteered to participate in interviews to schedule virtual meetings.

### Design

In 2023, the research team, consisting of four individuals from diverse and multiple backgrounds (informatics, qualitative research, quantitative research, psychology, and medical education), developed an interview guide. The interview guide included questions regarding demographics (eg, professional background, department or specialty, and patient population), innovation of CP/CF, CP/CF use, factors influencing use such as environmental pressures, types of consequences, and the future of CP/CF (Multimedia Appendix 1). Each interview was conducted by 2 team members virtually through Cisco Webex (one interviewer was the lead, who asked the questions on the interview guide, and the second interviewer asked follow-up questions as well as any questions that may have been missed by the lead interviewer). Interviews were recorded and professionally transcribed.

### Analysis

In 2024, the research team used both a thematic deductive framework, deriving themes from existing theories, and a thematic inductive framework (where themes are derived organically from raw data) to analyze data. Two team members used 3 transcripts to develop the initial codebook. Five team members then analyzed all 22 transcripts, consisting of 322 pages of data, using a standard inductive grounded approach, iteratively developing a codebook, and generating patterns and themes. NVivo 12 (Lumivero) was used for the management and analysis of the data. The inductively generated themes naturally fell into categories described in the Diffusion of Innovations Theory of Unintended Consequences [22], so this framework was applied to describe the results.

## Results

### Overview

Findings are organized using the Diffusion of Innovations Theory of Unintended Consequences framework in Figure 1, enhanced by inductively produced themes. Rich quotes from interviewees are available in Multimedia Appendix 2.

### Demographics

In 2023, we conducted 22 interviews (mean duration 48 minutes, range 29 to 65 minutes). Half were faculty (11/22, 50%), and most treated adult or family populations (17/22, 77%; Table 2). Most respondents were specialists (14/22, 64%). Many of the respondents saw patients in both inpatient and ambulatory settings (10/22, 45%), with some respondents reporting seeing patients in ambulatory settings alone (8/22, 36%).

**Table 2:** Demographics table: qualitative interview respondents’ characteristics, including role or training level, population, department, and setting.

Respondent characteristic			Value, n (%)				
**Role or training level**							
	Faculty	11 (50)					
	APP^a^	7 (32)					
	Resident and fellow trainee	2 (9)					
	Medical student	2 (9)					
**Population**							
	Pediatrics	5 (23)					
	Adult and family	17 (77)					
**Department**							
	Specialists^b^			14 (64)			
	Primary care, family medicine, and internal medicine	6 (27)					
	Students			2 (9)			
**Setting**							
	Inpatient	4 (18)					
	Ambulatory	8 (36)					
	Both	10 (45)					

^a^APP: advanced practice provider (physician’s assistant, nurse practitioners, certified nurse, and licensed social worker).

^b^Specialists include 1 each of the following: emergency medicine, women’s health, pediatrics, urology, midwifery, dermatology, psychiatry, rheumatology, neuroradiology, infectious disease, sepsis, neonatology, clinical informatics, and hematology-oncology.

### Innovation

The model (Figure 1) starts on the left with the innovation. The innovation is a new idea: in this case, duplicative text tools in the EHR. Respondents believed that because the documentation burden was so high for providers, CP/CF were developed as innovative solutions. Textbox 1 depicts the themes and subthemes found in the results.

**Diffusion of Innovations Theory of Unintended Consequences Framework components from Figure 1 and the subthemes related to each of the framework components (subthemes are displayed as bullet points).**

Framework component (from Figure 1) and subthemes

**Innovation**

**Diffusion of CP (copying and pasting)/CF (copying or carrying forward) use**

**Communication (influences)**

**Social system**

- Overview
- Differences in types of patient visits
- Difference in the health care delivery system
- Difference in specialty
- Difference in electronic health record (EHR) products

**Intended consequences**

- Overview
- Efficiency or decreased documentation time
- Organization and time management skills
- Increased communication
- Improved accuracy for objective data

**Unintended consequences**

- Overview
- Inaccuracies and errors in the note
- Plagiarism
- Note bloat
- Critical thinking issues
- Ultimate unintended consequence: inner angst or moral injury

**Outside environment**

- Overview
- EHR-related solutions
- Artificial intelligence–related solutions
- Innovation: people-focused solutions
- Cultural shifts in documentation

### Diffusion of CP/CF Use

Moving to the center square (Figure 1), consideration of the diffusion of CP/CF includes (1) how people adopt tools, (2) why people use these tools, and (3) why some decide not to use these tools.

In the case of CP/CF, some respondents noted that at times, documentation duties occasionally fell onto the residents/fellow trainees and APPs, so these tools are necessary for efficiency purposes. Residents/fellow trainees are, at times, active change agents in the diffusion of CP/CF because “it would be impossible for trainees to not copy and paste” (faculty). However, residents/fellow trainees are not the only ones who document and use CP/CF. When faculty are not supervising residents/fellow trainees, they are writing notes themselves and use these tools regularly, so they can achieve a better work/life balance.

### Communication Influences

The superior aspect of the model (Figure 1) indicates that communication impacts the diffusion and spread of an innovation (ie, CP/CF). Information about CP/CF was commonly communicated through informal training and education from other colleagues rather than through formal training. Medical students noted that communication about the appropriate use of these tools was inconsistent. Some direct supervisors (ie, resident/fellow trainees) vocalized and encouraged medical students to use the tools and “start building [these skills now]” (medical student), while others (ie, faculty supervisors) did not want them to use the tools because, as students, they needed practice writing notes themselves and developing critical thinking skills.

### Influence of Social Systems

#### Overview

The inferior aspect of the model (Figure 1) indicates there are societal or systems pressures influencing how and when CP/CF are used. Use of CP/CF varied greatly by type of patient visits, setting, specialty, and attributes of the EHR product.

#### Differences in Types of Patient Visits

The use of CP/CF differed depending on the type of patient visit (eg, telephone, virtual, or in-person visit; returning, new, or old patient). With a new patient, providers reported using CP to gather the patient’s historical information, especially when the history was complex and involved many images. However, there were some notable differences in use among clinicians. For example, a surgical resident/fellow trainee noted that he was more likely to write down verbal history when meeting a patient for the first time versus gathering prior information and using CP. Other providers mentioned that they do not use CF because they view each visit as a new visit.

#### Difference in Health Care Delivery Setting

According to our respondents, CP/CF use is influenced by whether care is provided in an ambulatory or inpatient setting. For example, CF has been described as “the backbone of all (in-)patient charting” (APP) because it aids in the continuity of care between team members, which would not be applicable in ambulatory settings. Providers who work in both inpatient and outpatient settings mentioned using CP in both settings because they are used to doing so.

#### Difference in Specialty

Respondents described how different workflows and documentation requirements impact CP/CF use. When providers see long-term patients for chronic problems, they rely on CP/CF, but if they consistently see new patients, such as in emergency medicine, they tend not to use them.

Specialties vary in how they structure their notes, impacting the value of CP/CF. Those with more structured and templated notes used CP/CF less frequently, whereas other specialties required documentation of detailed thought processes and thus were less structured and used CP/CF more frequently. Volume and duration of the patient visits differed by specialty, which impacted providers’ use of CP/CF, with one faculty member noting, “when I’m busier, I tend to copy/paste more and copy forward more” (faculty). The research team also noted that the value found in using CP/CF differed by specialty, so developing a single policy to guide CP/CF throughout a health system would be challenging.

#### Difference in EHR Products

EHRs vary based on product offerings and integration into a system, including access to and policies for use of duplicative text tools. One resident/fellow trainee compared one academic center’s commercial EHR to the Veterans Affairs’ EHR, noting that CP is not easy to do, and CF is not available at all.

### Intended Consequences of CP/CF

#### Overview

The right aspect of the model (Figure 1) features the intended and unintended consequences of implementing any new technology. Our analysis revealed intended benefits from the innovation of CP/CF tools.

#### Efficiency or Decreased Documentation Time

When asked about the benefits of CP/CF, many respondents highlighted that these tools were “a survival mechanism” (faculty) that streamlines work processes, decreases documentation time, and increases efficiency, which reduces provider stress and promotes work/life balance.

#### Organization and Time Management Skills

Part of the CP/CF’s efficiency is derived from organization and time management, allowing providers to update relevant data in a template rather than recreating an entire note from scratch. When used this way, CF can provide consistency, organization, and standardization of notes, helping providers find salient information quickly.

#### Increased Communication

Respondents discussed CP/CF’s enhancement of communication within and across teams and disciplines and settings, which “saves [them] a great deal of time” (faculty). CP/CF help providers stay organized in their notes, which allows them to solve problems together.

#### Improved Accuracy for Objective Data

CP/CF may decrease errors in translating complicated or nuanced data from note to note, especially from specialists or when dictating notes. One example noted was radiology, because the integrity and accuracy of the original report should stay intact, and providers “don’t have translational[/dictation-relations] errors” (faculty).

### Unintended Consequences of CP/CF

#### Overview

While innovations bring about intended and desirable consequences, unintended consequences can also affect the adoption of innovations (Figure 1). Although the diffusion of CP/CF into EHR systems could be considered a success, respondents discussed the risks and challenges CP/CF use presented, which shaped their willingness to use CP/CF in their practice.

#### Inaccuracies and Errors in the Note

All respondents noted that CP/CF use can increase inaccuracies and errors in the notes, from the seemingly innocuous errors (spelling errors) to harmful errors (forgetting to update a note). Information can be outdated yet copied. Errors can be perpetuated. Respondents admitted to forgetting to update information they had previously copied, especially in hectic environments. Some respondents expect that inaccuracies and errors will occur due to the “amount of pressure they feel to get [notes] done” (faculty) and the pressure on providers to see higher volumes of patients.

Whether inaccuracies are accepted as part of the cost of using efficiency tools, these errors can be harmful, in some cases leading to legal as well as patient safety risks. One risk is putting the copied text into the wrong chart, allowing patients to see other patients’ confidential information. Another risk is carrying forward a care management plan that may not be appropriate for patients. One faculty member noted, “whatever we are signing our name [to] should be our own work” (faculty), and that signing off on a note that they have not reviewed thoroughly is problematic.

#### Plagiarism

Respondents noted that plagiarism can be another unintended consequence of using these tools. Attribution was another issue, with respondents discussing how they, or others, copied from other people’s notes without citing where they obtained this information or verifying that the information they copied is correct.

#### Note Bloat

One of the most cited challenges with the CP/CF innovation was the increased length of notes or note bloat. Note bloat makes quickly finding relevant or updated information within a large volume of data more challenging. The length of notes also contributes to the perpetuation of errors. As described by respondents, CP/CF contribute directly to note bloat. Additionally, longer notes mean that providers may not be reviewing and editing notes as carefully as they should, allowing errors to slip through.

#### Critical Thinking Issues

Respondents described how the use of CP/CF affected critical thinking skills and led people to not review information thoroughly. Providers, especially medical students and residents/fellow trainees, must learn how to think critically. As one faculty member noted, providers must “separate the wheat from the chaff” (faculty) and determine what to include in a note; critical thinking is an important part of training, which may be impeded by shortcuts in documentation.

#### Ultimate Unintended Consequence: Inner Angst or Moral Injury

The constant friction between the internal and personal desires to practice caution, review information thoroughly, and create concise and accurate notes, and the external pressures of practicing medicine in today’s organizational environment, has led to the most important unanticipated and unintended consequence of the CP/CF innovation: a sense of inner angst, or moral injury, among respondents. The concept of medical moral injury (or distress) among providers has gained traction in the literature in recent years, and covered actions due to commission (providers acting against a belief) or omission (providers not able to act on their beliefs), whether due to a sense of being internally or externally constrained in their ability to provide what they believe is quality care in a high-stakes situation or betrayal from people, institutions, or systems in power [24]. Whether conscious of these feelings of inner angst or moral injury, respondents discussed at length the impact on and of CP/CF use.

Documentation burden, due to time constraints and increased pressure to see higher patient volumes, negatively affected providers’ sense of well-being and created pressure to use these tools, even if they did not want to do so. With a higher patient census, documentation requirements increase, as does the amount of time providers need to review other people’s notes. Due to these conflicting issues, the compromise between efficiency and accuracy is a challenge for providers. Respondents described a love/hate relationship with CP/CF, in that, despite the risks, the tools are necessary to function in the current environment.

Despite the acknowledgement that CP/CF can create some efficiencies, many respondents called CP/CF a “necessary evil” (APP) due to the risks and challenges arising from use.

Respondents experience this internal struggle with wanting to be thorough, efficient, and accurate with documentation, but they do not have enough time to document, so they use tools that they know have errors associated with them, with some calling CP/CF “sloppy and paste” (faculty). Respondents feel this inner angst or moral injury because they have no other choice when the institution is pushing them to see more patients without allocating more time or compensation to document.

### Influence From the Outside Environment

#### Overview

Further complicating and adding to the inner angst experienced by providers, the outside environment influences diffusion of an innovation and its consequences (Figure 1). Not only are providers feeling internal pressures, but they are also experiencing outside influences, including legal, regulatory, and financial requirements regarding what content to include in the note. This pressure, and the possible consequences, directly impact how respondents use CP/CF. In one example, a faculty member noted that the Centers for Medicare and Medicaid Services require an enormous number of requirements for one note, with some calling the note the “patient Wiki[pedia]” (faculty) for how many purposes the note serves at one time.

#### EHR-Related Solutions

Respondents provided suggestions for improving the EHR infrastructure to increase efficiency, including smart phrases, as well as improving education on EHR use. Part of that education includes understanding what information is relevant to keep in the notes to increase the usefulness of currently available efficiency tools, whether through CP or smart tools (Epic Systems). Respondents also expressed a desire for dictionaries that autocorrect abbreviations or prepopulate problem-based notes.

Another suggestion was for a tool that automatically identifies whether any information had been copied, with author attribution prominently displayed, such as a pop-up alert when using or reviewing text created with CP/CF and requiring users to review common errors from CP/CF, such as dates or values, before closing an encounter*.* Respondents also mentioned needing alternative tools to display and integrate notes, as well as more standardization and guidelines in note creation. Others suggested having some sections of the notes be collapsible, as well as being able to insert images and hyperlinks within a charting tool for easier access to other areas of the chart.

#### Artificial Intelligence–Related Solutions

Some respondents described recent advances in artificial intelligence (AI) and the potential for integration into documentation tools. They wished that the note could be automatically created “like Jarvis style from the Avengers” (faculty). One such advancement currently under investigation is the use of ambient scribes, which listen to the physician-patient encounter and write the notes on behalf of the providers.

#### New Innovations: People-Focused Solutions

As much of the training and communication in EHR use to this point has arisen from informal communication channels (eg, word of mouth) or piecemeal, based on workflows and department needs, respondents requested additional education and training on best practices for documentation. Respondents also suggested training should focus on what the “right tools are to do the work” (APP), which could minimize reliance on CP/CF as the primary documentation tools.

Equally critical to providing training on best practices in documentation is giving providers the amount of time necessary to set up templates and smart phrases in the EHR, as well as an understanding of what is important to include in a note, ensuring documentation is more efficient in the future. Respondents proposed new or updated guidelines and standards with clear teaching and expectations of use for CP/CF to ensure efficient use of the tools. Departmental guidelines and expectations surrounding CP/CF use could help new learners and faculty use these tools appropriately.

The creation of a super user role on teams to facilitate general knowledge-sharing could provide a point person to identify key workflow components and functionality within departments or systems, which could “eliminate the amount of overload that the Epic support team [gets]” (APP) and lessen prolonged waiting times for users.

#### Cultural Shifts in Documentation

One of the key barriers to reviewing and editing notes for conciseness and accuracy is time, where *“*[time spent on editing the notes] is seen as an extra burden” (resident/fellow trainee). This extra time often is uncompensated and means less time caring for patients. The time spent on editing notes has unanticipated consequences of external constraints (eg, reimbursement policies or patient volume targets) on inner angst. An APP noted she wished she could spend more time with patients and less time documenting in general. Another APP highlighted that, “there’s been such an increasing pressure to see more and more patients… and that’s just making it harder and harder to actually document” (APP). With the current documentation culture, one resident/fellow trainee noted that, “the only way to function really, is to be copy and pasting from yourself [and] from others” (resident/fellow trainee).

Providers argued that there needs to be a cultural shift with regard to best practices for what gets documented and put into the note, as well as how notes are used. The reliance on notes as a patient “Wikipedia” has contributed to note bloat and the rise in errors and inaccuracies, as well as provider burnout, and therefore, reliance on CP/CF to document quickly. Providing the time needed for reviewing the work is critical.

Some providers suggested that the use of these tools should be limited. One faculty member said attribution should be mandatory; others added that the copied portions should be cited in quotes and attributed to the original author as in peer-reviewed publications. It could also be helpful to use different fonts, italics, or bold typeface to indicate copied text. Others mentioned that CP/CF should only be used for what is necessary to contextualize or augment the note, with clear identification of the information’s source.

## Discussion

### Summary

In this study, we identified perceptions of and attitudes toward CP and CF, as well as consequences of the diffusion of this innovation in note writing, in an academic hospital (Figure 1). Whereas prior studies have described provider perceptions and process improvement through the introduction of CP/CF, our research expands on this literature by qualitatively exploring the consequences of this innovation through the lens of unintended consequences in diffusion [19] (Textbox 2).

**Summary findings are split up by the components of the Diffusion of Innovations Theory of Unintended Consequences framework from Figure 1.**

**Communication (diffusion)**

- Documentation burden falls greatly on providers—but with pressures on their time, diffusion (via communication or training) is limited and inconsistent—users have created ad hoc workarounds to deal with the workload, versus intentional evidence-based practice and critical thinking skills development.

**Social system**

- Note purpose and use vary by specialty, health care setting, patient visits, and EHR products, so a one-size-fits-all solution is not feasible.

**Intended consequences**

- Respondents report a more streamlined workflow and increased efficiency, especially those with templates to help with organization and consistency, and can help with inter- and intrateam communication—when used appropriately.

**Unintended consequences**

- Whether through limited training, anxiety around policies, or other challenges, inaccuracies in notes result in risks to the patient, author, and system, from simple to severe. Respondents experience the moral injury of spending more time documenting out of fear of liability, harm, and denial of reimbursement, versus spending time providing care to patients, changing the culture of care. Noat bloat increases, the culture changes, and consequently, users experience frustration and burnout.

**Outside environment**

- Legal, regulatory, and financial pressures require documentation, but users are having to adjust their workflow to the requirements, rather than the system adjusting to the workflow. Respondents hope innovations will help with efficiency, but question where they will fit in the current external environment, which is perceived as broken, or whether they will arise from within. There is a need for guidance and policy, but it is hard to do so when use is so variable.

### Discussion of Framework Elements

#### Communication

Awareness of CP/CF tools spread by word of mouth (Figure 1). As reported by others, the diffusers (ie, the ones spreading the innovation) are sometimes residents/fellow trainees and APPs, rather than supervisory or more senior staff, a trend that has persisted over the years [8,20,21,25]. However, simple awareness does not guarantee the quality of the information communicated in the medical note, so tailored formal training is recommended [26,27]. Another recommendation to promote high-quality training is to have an EHR champion available, especially in the inpatient setting where CP/CF are most used [8,20,27,28].

Communication and training about the use of CP/CF in documentation can provide teaching opportunities to enhance critical thinking of relevant information to capture [29]. This may be less likely in high-pressure, dynamic systems, but some have had success in training through templated or team-based writing [29,30].

#### Social System

Innovations (Figure 1) such as CP/CF are highly complex and are integrated into similarly complex and evolving systems [22,31,32]. Implementation and adoption rely not only on individuals and their personal characteristics, but the innovation itself, as well as the structure and characteristics, and culture of the organization in which the innovation is deployed. By certain metrics, innovations may be successfully diffused (technically adopted), but a lack of consideration for the interdependency of the actors in a system may inhibit realization of the optimal or true value to the system, and can lead to unintended consequences [31,32].

#### Intended Consequences

Our results reflect those of other reports that CP/CF improve efficiency [6], continuity of care for patients [8], and communication with teammates [33], as well as decreased documentation burden [7]. Yet in the two decades of literature acknowledging the benefit of innovation in electronic records, these same reports highlight the double-edged sword of efficiency versus unintended consequences [8,34]. Without trust in the system or other users, or without bulwarks against our own inherent flaws as human users of technology [35], the optimal use and benefits of these innovations and the intended consequence of improving quality of care will not be realized [31].

Our respondents describe the need for short-term solutions, such as recurring formal training on how and when to use CP/CF. Others have similarly demonstrated that lack of knowledge of appropriate use results in increased length of notes [36] and the importance of training and education for safe practice regarding these tools [31].

#### Unintended Consequences

Documentation burden, note bloat, inaccuracies, and more time spent documenting to the detriment of patient care, training, and interpersonal communication and trust—all have led to a perceived worsening of culture, the work environment, and workflows [5,37]. These phenomena are not new; the same benefits and challenges have remained consistent over time and across systems [38-40]. This dichotomy of intended (often positive) consequences and unintended (often negative) consequences results in providers experiencing increased inner angst and moral injury [41,42]. Providers report angst from constraints against practicing what they consider “quality” care, and despite knowing the myriad risks, use tools such as CP/CF as the only workaround right now in a broken system, calling CP/CF a necessary evil. While providers acknowledge that CP/CF and other tools aid in the continuity of care and create a sense of efficiency when documenting, the lack of time to review notes thoroughly is a consistent and pervasive structural barrier. In such instances, errors slip through the cracks—the role of CP/CF in errors, inaccuracies, and outdated information is of particular concern [6,12], as they have contributed to note bloat [5,8] and may play a role in patient safety [16]. Inaccuracies may damage a patient’s trust in and experience with the clinician or system; contribute to diagnostic and safety errors (inaccurate or out-of-date records, difficulty in finding relevant data in bloated notes); and increase legal risks, including unintentional falsification of records or plagiarism and medical malpractice [16,17]. All of these factors conspire to reduce providers’ overall perceived quality of care delivery. This, in turn, can further worsen provider dissatisfaction and burnout, with a study suggesting that clinician involvement in medical errors is an independent risk of burnout [43].

#### Outside Environment

The outer system (Figure 1) is a key factor for optimal diffusion and implementation of an innovation [32,39]. Recommendations to address challenges in using CP/CF include developing policies, both institution-wide and specialty-specific, for CP/CF use. These larger structural factors—policies, standards, legislation, system resources, and engagement of leadership—filter down into organizational workflow and processes and then to individual knowledge of the innovation [32,39,40]. Although improvements at this larger scale are difficult to accomplish and are often more long-term solutions, a fundamental shift is needed at the outer system level in how these innovations are built for and integrated into dynamic, complex systems. Notes should serve clinical purposes and not focus as much on reimbursement regulations and national quality measures [21,32,44].

#### New Innovations

In the short term, most providers report the need for specific interventions, such as training to improve optimal EHR use, continued EHR optimization, and improved awareness of best practices for use of the functionality are necessary. Others recommended some degree of restrictions on use as well. While shown in other health systems to have direct impacts on hospital readmission [45], the overall widespread reliance on its use, and the inability to remove this functionality (easily) from underlying operating systems, suggest this is a nonviable solution. As a result, one must look for more novel, sustainable solutions. With the growth of AI and tools such as ambient or digital scribes already in use in clinical care to reduce documentation burden, careful reflection on the appropriate use of innovative tools will be paramount in mitigating current and future unintended consequences [6,13,46-48]. Another way to mitigate unintended consequences for ambient scribing would be to develop measures and metrics of success (eg, provider satisfaction scores, decrease in pajama time charting, decrease in documentation time, and increase in work/life balance). That way, organizations could evaluate whether the ambient scribes are aiding providers related to the intended metrics. However, while ambient scribes hold promise, to date, no studies have documented the impact of the ambient scribes on the amount of CP present in notes. This is likely, in part, because studies on ambient scribes are limited to ambulatory settings [49]. This will need to be an area of future study as the technology becomes more widespread.

### Strengths and Limitations

There were some strengths in our study. First, this paper addresses providers’ perspectives toward CP/CF in a way that adds depth and insight to the body of literature already present. Second, this is one of the first papers that attributes the concept of moral injury to the use of CP/CF, which has not been explored in the literature. Third, this manuscript offers recent perspectives and alternative solutions to aid in the documentation burden (eg, AI scribing). Fourth, this paper provides a true direct negative harm of CP/CF to the users, something which has not been previously described. In essence, while CP/CF use is continuing to grow over the years, it is associated with higher rates of documentation burden and job dissatisfaction. Thus, it is not working to solve the primary problem it was adopted to address: to make providers efficient and alleviate documentation burden. One reason for this may be the unintended consequence of its use, that whatever benefit the provider obtains from using these tools, moral injury and guilt compound and hinder providers.

There were some limitations to our study. First, this research was conducted at one academic health care institution, which may limit the generalizability of the findings. However, we solicited a breadth of input from various departments, roles/training levels, and clinical settings. Further, reported results from previous research on CP/CF are consistent with our findings, suggesting that these phenomena go beyond our institution. We also engaged a diverse team of researchers from a variety of disciplinary and demographic backgrounds to analyze the data to ensure a multidisciplinary approach and perspective. Second, it should be noted that the respondents for interviews were recruited from a subset of respondents to a previously deployed anonymous survey [19], specifically those who had self-identified and volunteered to participate in interviews. This approach does raise the likelihood of amplifying potential selection bias. Additionally, we used a convenience sampling method to select participants for interviews, and while the initial survey used a more robust purposive sampling strategy, the subset of self-identified interview participants was predominantly faculty and APPs, as opposed to medical students and residents/fellow trainees. As the use of CP is ubiquitous across levels of training and professional experience, we believe that our sampling strategy does reflect a broad and adequate spectrum of perspectives despite the relative underrepresentation of medical students and residents/fellow trainees. Third, at the time these interviews were conducted, there had been some discussion within the institution about whether curtailing or banning the use of CP/CF was appropriate as a policy measure. However, despite this potential barrier, it should be noted that respondents uniformly expressed both positive and negative perceptions of EHR documentation tools.

### Future Research Directions

Research addressing the unintended consequences of CP/CF, such as the use of a note for multiple purposes (eg, legal and patient safety implications, billing, communication, and continuity of care), could lead to slimmer, more streamlined notes, a decrease in documentation burden, and less burnout.

While CP/CF has limitations, there are innovations that could encourage appropriate use or reduce the need for CP/CF in documentation. Some potential innovations discussed by our participants were EHR technical solutions, including automatically identifying information that has been copied and having pop-up alerts when using or reviewing texts. Future research on the role these proposed features have in documentation could inform additional technical innovations and support cultural practices for proper attribution. AI solutions, such as digital or ambient scribes, may decrease the documentation burden and the need to use CP/CF; research is ongoing [46,50,51], but future research is needed to understand use across systems with different EHR policies and capacities. People-focused solutions, including more robust orientation training on CP/CF and having staff EHR super users to facilitate general knowledge sharing, could support more efficient and appropriate use of EHR tools, which in turn could help address documentation burden. Future research of educational programs and inclusion of EHR super users pre- and postimplementation may provide justification to integrate these ideas into clinical practice workflows, supporting a culture valuing time for training. Furthermore, importantly, health care systems are complex environments, and implementing new tools can result in unintended consequences. Future work must consider the potential for these unintended consequences resulting from the implementation of a complex innovation in dynamic systems.

### Conclusions

Our study aimed to use semistructured interviews with providers to investigate their perceptions and attitudes surrounding CP/CF in the EHR, using a modified Diffusion of Innovations Theory of Unintended Consequences framework to analyze the data. The intended consequence of the innovative CP/CF tool, efficiency, is valued by users. However, the unintended consequences, including note bloat, errors, and patient safety and legal risks, have given rise to moral injury, a finding consistent with the literature. The theory of unintended consequences framework addresses the complex system within which CP/CF exists. In the case of CP/CF, outside pressures on documentation requirements are one of the critical barriers to realizing the potential for innovative duplicative text tools. Fewer documentation requirements may lessen internal and external pressures, leading to inner angst or moral injury among providers.

## Appendix

Multimedia Appendix 1 Participant interview guide.

Multimedia Appendix 2 Table of quotes from participants in relation to the diffusion of innovation themes.

## Abbreviations

AI: artificial intelligence
APP: advanced practice provider
CF: copying or carrying forward
CP: copying and pasting
EHR: electronic health record
VistA: Veterans Health Information Systems and Technology Architecture

## Data Availability

The dataset generated or analyzed during this study is available from the corresponding author on reasonable request.
