# Supplementary material for: Copy Tools in the Electronic Health Record: Perceptions, Implications, and Future Directions
Source: JMIR Med Inform. 2025 Dec 19;13:e78502. doi: 10.2196/78502 (PMC12759298; doi:10.2196/78502)
Supplement: Multimedia Appendix 1 [file medinform_v13i1e78502_app1.docx]

Copy/Paste and Carry Forward Interview Guide

For Med Students/Faculty/APPs/Residents and Fellow Trainees

Interviewee’s name: __________________ Interviewer’s name:_________________________

Date: _____________ Time:___________ Setting:_____________

Recorder’s name:____________ Folder name/#:_____________ Record #:___________

**Set phone etc. to silent!**

Introduce self and assistant. I will conduct the interview but assistant will have a chance at the end to ask follow-up questions. Talk about why we are conducting this study and emphasize that we are not trying to take this functionality away.

Refer to the information sheet explaining the study and ask for questions. Note that you will be asking for consent once the recording starts.

**Turn on recorder**

It’s [date] and this is [X and X] interviewing [Y] at [site name]. We have reviewed the Information Sheet, and would like to record this interview, and we need your verbal consent- do you agree to being recorded?

In this interview, we will be discussing your use of copy/paste and carry forward in the EHR and discussing possible solutions to make those functions better. Our goal is to enhance, not inhibit, their use. For our purposes, when we ask questions about copy/paste, we are defining it as, when you select certain parts of a past note and just copy that part of it. We are defining carry forward as when you carry forward the whole note and then edit it for today’s visit. For our purposes, templates and dot phrases are not considered part of copy/paste or carry forward.  And all further questions in the interview will pertain to your experiences at X and X’s version of Epic. We will start this interview by asking you some demographic type questions.

1. **Demographics**
2. First, we’d love to learn a little bit about you. Could you tell us about your background, role, and professional training?
3. Probe: in what settings do you frequently work (out-patient, emergency department, inpatient primary care services, inpatient consult services)
4. Probe: How long have you been at X?
5. Probe: if they mention that they have been a scribe in the past, ask them: what was your use of copy/paste and carry forward like as a medical scribe?

1. **Your Use of Copy/Paste**
2. How much do you use copy/paste?
3. In terms of percentages, roughly what percent of your note do you think is copy/pasted?
4. Probe: How different is the percentage depending on what care environment you work in (for example like comparing working in clinic vs working in the ED)
5. Probe: How?
6. Probe: Why?
7. How much does your use of copy/paste fluctuate from day to day or during any individual day? (For example, if you have a busy clinic or a large patient load, etc.)
8. How much has your use of copy/paste increased or decreased over time as your role has evolved?
9. Probe: Why?
10. What sort of training did you receive, either formally and/or informally, on how to optimally use copy/paste in your notes?
11. How has the covid19 pandemic effected your use of copy/paste?
12. Would you please describe how your use of copy/paste fits into your general workflow (i.e. how much does your use of copy/paste complement your care team (providers, residents/fellows, and medical students))?
13. How much do you think you use copy/paste compared to your peers/people at your same level?
14. How much copy/paste do you use compared to others in your care team?
15. If medical student, compared to residents and faculty
16. If resident, compared to faculty and med students
17. If faculty, compared to med students and residents
18. What information do you copy/paste?
19. Probe: And for what parts of the note is copy/paste most valuable? (Like imaging, radiology, etc.)
20. How much do you copy/paste from other people’s notes versus your own notes and can you tell me more about that? (For example, whose notes do you copy/paste from? what kind of notes are you copying from? For example, are you copying from med students, residents, fellows, providers, or yourself?)

1. **Risks/Benefits of Copy/Paste**
2. How do you know when others have used copy/paste in a note?
3. Have you noticed any errors being made while using copy/paste? Either your own or someone else’s errors
4. Probe: In what context have you noticed these errors?
5. Under what circumstances would you **not** use copy/paste?
6. What kind of feedback have you received because of your copy/paste use? Or do you find yourself talking to other people about copy/paste?

1. **Onward and Upward Questions: Copy/Paste**
2. In the survey, we found that people like using Copy/Paste but also understand that there are risks associated with it. In a perfect world, how could things related to copy/paste be better?
3. In your opinion, what would be a better solution than copy/paste?
4. Why do you personally choose to use copy/paste for your notes?

1. **Carry Forward or End Conversation**.
2. In your opinion, is there a difference in how you use carry forward compared to copy/paste?
3. If yes: please ask the participant to describe the differences and move on to the carry forward questions
4. If no: Ask this question: We very much appreciate you taking the time to participate in our interview. Do you have any other questions you would like to ask us? Or anything else you would like to share regarding your thoughts and feelings around copy/paste and carry forward?

1. **Your Use of Carry Forward**
2. How much do you use carry forward?
3. Does your use of carry forward depend on what care environment you work in (for example working in clinic vs working in ED)
4. Probe: How?
5. Probe: Why?
6. How much does your use of carry forward fluctuate from day to day or during any individual day? (For example, if you have a busy clinic or a large patient load, etc.)
7. How much has your use of carry forward increased or decreased over _____ (time will be relevant to their role)?
8. Probe: Why?
9. What sort of training did you receive, either formally and/or informally, on how to optimally using carry forward?
10. How has the covid19 pandemic effected your use of carry forward?
11. Would you please explain how your use of carry forward fits into your general workflow (i.e. how much does your use of copy/paste complement your care team (providers, residents/fellows, and medical students)?
12. How much do you think you use carry forward compared to your peers?
13. How much carry forward do you use compared to others in your care team?
14. If medical student, compared to residents and faculty
15. If resident, compared to faculty and med students
16. If faculty, compared to med students and residents
17. After you have carry forwarded a note, what are the common elements of the note that you change?
18. How much do you carry forward from other people’s notes and can you tell me more about that? (For example, whose notes do you carry forward from? what kind of notes are you copying from? For example, are you copying from med students, residents, fellows, providers, or yourself?)

1. **Risks/Benefits of Carry Forward**
2. How do you know when others have used carry forward in a note?
3. Have you noticed any errors being made while using carry forward?
4. Probe: In what context have you noticed these errors?
5. Under what circumstances would you **not** use carry forward?
6. What kind of chatter from others have you heard about regarding carry forward? Whether that be feedback you have received regarding copy/paste or conversations you have had or heard from others.

1. **Onward and Upward Questions: Carry Forward**
2. In the survey, we found that people like using Carry Forward but also understand that there are risks associated with it. In a perfect world, how could things related to carry forward be better?
3. In your opinion, what would be a better solution than carry forward?
4. Why do you personally choose to use carry forward for your notes?
5. We very much appreciate you taking the time to participate in our interview. Do you have any other questions you would like to ask us? Or anything else you would like to share regarding your thoughts and feelings around copy/paste and carry forward?

1. **I’d like to give my colleagues a chance to ask some questions**
