# Supplementary material for: Copy Tools in the Electronic Health Record: Perceptions, Implications, and Future Directions
Source: JMIR Med Inform. 2025 Dec 19;13:e78502. doi: 10.2196/78502 (PMC12759298; doi:10.2196/78502)
Supplement: Multimedia Appendix 2 [file medinform_v13i1e78502_app2.docx]

Table of Quotes from Interviewees

| **Section of Paper Quotes Belong In** | **Quotes** |
| --- | --- |
| **The Innovation** |  |
| **Diffusion of CP/CF use** | *“It would be impossible for residents to not copy and paste because they have this high volume of documentation, because they are using their notes to present the patient. And if we said, ‘Listen, we really want you to sort of adopt more of a lean note, let us just write down what we are doing today with the patient,’ they would absolutely not be able to do their jobs” (Faculty).* |
|  | *“[CP/CF] is a really helpful way to keep information unified in a single place and going forward and making changes to it as they come as opposed to trying to reinvent the wheel every day, which I think would lose a lot of information” (Resident/Fellow Trainee).* |
| **Communication influences** | *“I would say to some of my colleagues, ‘How can you get out of clinic so timely?’ When I am here for an hour, to sometimes two hours, charting my notes and I cannot keep up. And they are like, ‘Have you not heard of copy paste?’ And I was like, ‘I was told never to use that button.’ And they said, ‘I do not know about you but I am not going to sit here and not get paid for charting for two hours.’ And I was like, ‘I would like to go home at night.’ And they said, ‘Copy and paste’” (APP).* |
|  | *“Some of the direct feedback we would get from rotation supervisors or clerkship directors would be, ‘Do not use it at all.’ And for a lot of the reasons that we have already talked about, right? There’s a lot of risks. It doesn’t always communicate the learner’s thinking, right? If an evaluator is trying to evaluate you based on your thought process and you are copy/forwarding someone else’s thought process, that is just not useful. It might be helpful for patient care but it is not helpful for evaluating you as a learner” (Medical Student).* |
|  | *“It was not a good day if the residents had to stay past 6 p.m., past sign out, and we were working on notes till 8 p.m. And copy/forward was what I saw everyone doing on the team, it was just their workflow, and as a student I felt like I was trying to replicate their workflow. The residents would often tell me just to go ahead and use [CF] to get it done quickly” (Medical Student).* |
|  | *“The interns and residents who were doing a lot of the [documentation] work were saying, ‘No, you need to start building [these] skills now’” (Medical Student).* |
| **Influences of social systems** |  |
| Influences of social systems: Differences in types of patient visits |  |
| Influences of social systems: Differences in healthcare delivery setting | “*I think [with] just about every inpatient setting and every specialty setting, CF is pretty regularly and aggressively utilized” (APP).* |
|  | *“I use copy forward on the inpatient side where we have a note that we’re updating day-to-day. I think CF has pretty much been the standard of practice for me across most of my inpatient teams and services and I think it has been overall a pretty helpful tool” (Medical Student).* |
|  | *“For inpatient, copy/forward is the backbone of all patient charting because you have so many team members that are going through this patient’s care and you have so many trainees that are starting notes and passing them off to other providers to finish them and so many co-authored notes. Copy/forward is something that everybody is depending on to be able to effectively chart in the inpatient setting” (APP).* |
| Influences of social systems: Differences in specialty | *“What we are seeing someone for is different every single time, and so while I do not have a strong desire to copy and paste, there is generally no point because we are doing something different all the time” (Faculty).* |
|  |  |
|  | *“I will work 7 to 14 days in the hospital straight, and on the first day of service, I do not have any notes for the day before. I will usually have a template in mind, so if I have a lot of time, I will pull in what I need and then craft an assessment plan. If I do nott have a lot of time, I will often times copy/paste from my previous colleague’s assessment plan and update it” (Faculty).* |
|  | *“In my environment, [CF is] heavily used primarily because it is a lot of resident teams [rotating] off and on. So, we had to establish, ‘This is the way we are documenting, this is the kind of thing that you need to put in there.’ And so, it is a service line. That was the way to keep things simple” (APP).* |
|  | *“There are certain services that are very structured in how they write their notes. And so, often I am not using copy/paste that much if the template is well structured and I do not have to pull in things on my own, like labs or imaging or go hunting down for certain things to include in the documentation. And then other services maybe where I am writing more of a free-hand kind of note, I think I would use copy/paste a little bit more in those settings” (Medical Student).* |
|  | *“I would say there's a pretty good amount of variability. I would say when I am busier, I tend to copy paste more and copy forward more. When my census is lower, I tend to copy paste and copy forward less” (Faculty).* |
|  | *“I know I have heard Dr. X talk and… he hates all copy/paste, and he sees no reason for a running history… but I think again, it’s very different documentation in the ICU and that’s very different from a rheumatoid arthritis patient over ten years. So, I think it is just the nature of the documentation really varies and that is why it is so hard to have hard and fast rules [around CP and CF]” (Faculty).* |
|  | *“I think notes would have to be a lot simpler or a lot more straightforward in how they are templated to justify not having copy/forward. Labor and Delivery is one that is a risk, where the notes are brief and they are very templated, and I can see in that case you would not have to use copy/forward because they are pretty quick to fill out. But on an inpatient team, like Internal Medicine or Family Medicine, where you have a lot of documentation around the thought process that has gone into patient care, that can be kind of hard to replicate on a day-to-day basis and that documentation is kind of the summary of several days of thinking about it. So, I think it would be hard to eliminate copy/forward [for the entire health care system]” (Medical Student).* |
| Influences of social systems: Differences in EHR products | *“At the VA, every computer takes about five minutes to log onto. And so, you cannot just say, ‘I am [going to] pop in here really quickly and do something.’ The medical record opens slowly. And then you cannot copy and paste easily, and you certainly cannot copy/forward. And so, every time you want to write a note for someone, you have to build it from the bottom up basically. It just takes a long time, time that could be spent providing patient care, learning to be a real doctor” (Resident/Fellow Trainee).* |
| **Intended consequences of CP/CF** |  |
| Intended consequences of CP/CF: Efficiency or decreased documentation time | *“You are able to take hours of work and use it and make adjustments as you need to make to still adequately provide care to somebody …without having to waste those hours again to recreate 90% of what already exists in the system” (APP).* |
|  | “*It’s just a survival mechanism, if we get five new patients overnight, it takes a long time to sit and kind of reinterpret and rewrite a lot of stuff” (Faculty).* |
|  | “*For a lot of people, a huge source of their stress associated with work is documentation and [documentation is the] source of burnout. So, they will do anything to make it more expedient to get that done” (Medical Student).* |
| Intended consequences of CP/CF: Organization and time management skills | *“I think the copy forward made it a lot easier to bring all the information to the next thing, refresh certain things with some of the hierarchy, or if you have a certain CBC you could refresh it, and it would kind of bring your new data in there” (APP).* |
|  | *“We are able to copy/forward our notes and just put our most up-to-date recommendations right at the top, so they do not have to worry that the format is changing” (Resident/Fellow Trainee).* |
| Intended consequences of CP/CF: Increased communication | *“[CP] saves me a great deal of time and it helps me communicate that to a multidisciplinary team in a succinct and thorough way” (Faculty).* |
|  | *“If someone else takes care of the patient, they know what we have tried and what has worked and what has not. That is what I find helpful. That is why I copy/forward and copy/paste because there is no way anybody is going to know that unless they go back to the last 15 office visits, which is very laborious” (Faculty).* |
| Intended Consequences of CP/CF: Improved accuracy for objective data | *“When you copy/paste, the advantage is you do not have translational errors. …You are not going to miss any[thing]. If you copy/paste a report of a radiology study, and it says, ‘Incidental granuloma of the chest,’ then it is right there in your note” (Faculty).* |
|  | *“I think in some ways, copying is safer than recreating the verbiage because you might mistype it or mis-speak it if you are using voice recognition. Or it might just not get recognized correctly” (Faculty).* |
| **Unintended consequences of CP/CF** |  |
| Unintended consequences of CP/CF: Inaccuracies and errors in the note | *“The most common thing that I see is typos. Somebody spelled the patient’s name wrong, and it gets copy/forward or copy and pasted into a section of a note” (APP).* |
|  | *“I’ve certainly gone back [and] seen something in a note that I was like, ‘Oh, man, that’s wrong.’ And I have signed it four days in a row, and I have to go back and adjust that because I did not notice it the first four days” (Faculty).* |
|  | *“I have seen and I have done so myself, where a provider will put that information in and say, ‘This visit.’ But you do not know what date that was. And so, when that gets carry/forward it, by default, makes an error” (APP).* |
|  | *“He said that he was reviewing a documentation meeting with a supervisor: and he just said, ‘Why is this patient getting a not very pleasant exam, every single day?’ And everybody looked at him and they said, ‘What do you mean?’ And he said, ‘Well, it says here that in the previous five days, you guys have performed this uncomfortable exam on this patient. Do you guys routinely do that every day?’ And they were embarrassed because they copied forward” (APP).* |
|  | *“Stuff that gets copy/pasted a lot are things like the history, especially if somebody is super slammed and you are on a consulting service. I have definitely seen people just copy/paste the E.D provider’s history and that is often not correct or there is often really important bits that were missed. And it is strange because usually I watch those providers ask the important questions in person, but then for some reason the note is sometimes just whatever was copy/pasted from the emergency department” (Medical Student).* |
|  | *“Comes down to the amount of care that people put into the documentation, the amount of pressure that they feel to get it done. I mean, if you look at what a primary care doctor has to do in the course of the day, and the number of notes that they have to write, there is extraordinary pressure on their efficiency and ability to document. So, it is not surprising at all that they would use these kinds of mechanisms and would occasionally make errors in correcting the note” (Faculty).* |
|  | *“There are other times where I have seen a note and it does not look like it has had any updates made to it at all, it has just been signed essentially with the exact same [information] as the day before. Mistake-wise, if you were trying to follow that note and make appropriate patient care decisions or give mediations, you could definitely make an error if you were to follow some of those things the way they’ve been written in the note…it is definitely a place where you could make medical mistakes and I have definitely seen outdated information especially get copy/forwarded” (Medical Student).* |
|  | *“The other thing that worries me a lot is when you copy/paste, you say you did things that you did not do… And that is falsification of your records, so copy/paste should be very clear that you copy/pasted” (Faculty).* |
|  | *“I firmly believe that whatever we are signing our name [to] should be our own work… [if you did not collect the history yourself], you should not be signing your name on there. And I honestly do not know if the citing name, date, and quotes is legally better or recommended.  But it sure as heck feels better because that is kind of what we do in academics in terms of like, ‘I am not claiming that I did this work.  But everything that is not cited, I am attesting that ‘I looked at it and that was the information that I got too’” (Faculty).* |
| Unintended consequences of CP/CF: Plagiarism | *“I hate copy forward because I think it is too risky, that we are going to miss things. I believe you should only copy and paste your own work, just like any other kind of educational endeavor” (Faculty).* |
|  | *“I think it is important to quote and attribute a text to the original author.  I would never just take from another author the text that they had written and plop it into my note. I mean, if that were done in any other context, it would be plagiarism, which is grounds for academic discipline” (Faculty).* |
| Unintended consequences of CP/CF: Note bloat | *“I would say overall [these tools are] overused. It leads to lengthy reports that duplicate a lot of information in other notes and other places in Epic” (Faculty).* |
|  | *“I observe some of my colleagues would copy/forward their assessment and plan from the last note to keep them updated as to what happened last and then they would say, you know, ‘From the last note, here is what happened.’ And then, today, and then they would write their blips for the day. The problem is the notes get very big when you keep doing that over and over again, and then that gets pretty hard to understand” (Faculty).* |
|  | *“Sometimes your eyes just do not see [the errors] and it is hard to be a super good copy editor when you are reading through previous notes. Especially when they are really long because then you are just bored by the time you are halfway through and you are really starting to skim” (Faculty).* |
|  | *“If you are going to do copy forward, then you have to comb through that note and remove things that are no longer relevant, but nobody has time for that. They just do not do that, and there is a ton of extra stuff [in the note]” (Resident/Fellow Trainee).* |
| Unintended consequences of CP/CF: Critical thinking issues | *“I think that again, just like all technology, we have to be careful in how we use it. I think that as people grow in their comfort in knowledge, I think they rely on it less and less because they do not need to feel like they have to keep using other people’s work. Like, they can think more for themselves. But I think especially for our trainees that are busy, it is so tempting to just keep doing it and I think they rely on it too much and as a teacher, it is hard to know what to teach if you do not know what they do and do not understand” (Faculty).* |
|  | *“I think you need to develop an art of copying what you think is important information and learning to separate the wheat from the chaff… I think it is easy to copy lengthy amounts of information that are useless. I think that is where the art of medicine comes in, is to know what is relevant and what is not” (Faculty).* |
|  | “*It is like telling your child, ‘Be careful. Do not do that. Make good choices.’ But I know that time constraints, emotional energy, and all the other things that people are doing within the hospital [make reviewing documentation] hard to do” (APP).* |
|  | *“I am not saying that people are doing horrible things, but it is easier to copy forward. You are not trying to do something bad, but you may not put as much care and attention into the documentation as you should” (Faculty).* |
| Unintended consequences of CP/CF: Ultimate unintended consequence: inner angst or moral injury | *“Copy and paste is helpful and it is harmful, it all depends on its intention” (APP).* |
|  | *“Physicians definitely feel like they spend an inordinate amount of time documenting. I feel that way, and I know a lot of my colleagues feel that way. I know that the residents spend an excessive amount of time documenting” (Faculty).* |
|  | “*I think you really start to see how copy/forward is really helpful to get things done in a time efficient manner so it is hard to imagine not having that option right now. I would hope that there would be some type of replacement for it or some other change that would make documentation time be as efficient. It feels like a pretty invaluable tool right now for teams to get things done in an efficient manner” (Medical Student).* |
|  | *“Unfortunately, [CP/CF is] a necessary evil in this environment [because] everybody is busy, and the system is what it is. There are all these pressures and documentation burdens and what people feel should be and needs to be in the document is challenging” (APP).* |
|  | One faculty member called CP *“‘sloppy and paste’* *because it kind of encapsulates this idea that oftentimes it is being used as an efficiency tool, but that it has the risk of spreading inaccuracies in the chart” (Faculty).* |
|  | *“If at some point in time, [the academic health institution] was like, ‘Okay, we cannot do any more copy forward, we cannot use these tools anymore,’ it would be a struggle for us” (APP).* |
|  | “*All of us have seen bad notes and been frustrated by reading bad notes. They see it as an efficiency tool and sort of an efficiency tool that can run amok pretty easily” (Faculty).* |
|  | *“It is much more efficient with the caveat that errors can be really big headaches. So, it is efficient if you do it correctly and if it works but if you make a mistake, it can be a big hangup that can actually take longer to unravel than if you just hand write your notes. But the problem is we do not have the time to handwrite all the notes all the time” (Medical Student).* |
| **Influences from outside environment: Overview** | *“They are using the note for four different reasons… They are always going to be shoving a ton of information into the notes and it is that untangling that is hard. They use it as a rounding tool at the hospital. They use it as the patient Wiki[pedia]… and then at some point, you are going to have to hand off to their doctor. It is just too much on one little progress note” (Faculty).* |
|  | *“There is a hesitancy and a fear of if you do not document every little thing. Then people say, ‘Oh, we are not going to bill effectively, we are not going to code effectively, we are not going to do this.’ And I think that puts a lot of pressure on people to use this workaround [CP/CF])” (APP).* |
| Influences from outside environment: EHR-related solutions | *“There is value in seriously considering some restructuring of our EMR’s infrastructure that would eliminate some of the barriers that lead people to use copy and paste” (APP).* |
|  | *“There would be value in reevaluating how we train [and educate] our providers in the EMR and really putting more emphasis on identifying what are the key components of their workflow that they truly need to be oriented on to be able to effectively utilize the tools and resources within our EMR that is available to them” (APP).* |
|  | *“There also would be, in a perfect world, more robust auto templating tools that formatted and collated information from the EHR in a way that was useful.  A lot of the auto templating things just pull in massive amounts of text in very poorly organized blocks that I think many of us do not use, because it makes the notes uninterpretable, and just so long” (Faculty).* |
|  | *“I hate copy forward because I think it is too risky that we are going to miss things. I believe you should only copy and paste your own work just like any other kind of educational endeavor, and as someone in a bunch of educational roles I have clearly tried to teach our learners that too” (Faculty).* |
|  | *“[wished the EHR could] collate things well and be able to automatically integrate different information so that you would not have to go and build this representation of who your patient is, and what you are doing for them. And I could focus just on the unique parts that I am providing as a physician, which seems like a vanishingly small part of our notes these days” (Faculty).* |
| Influences from outside environment: Artificial intelligence (AI)-related solutions | *“If I had a genie and could have any wish I wanted, I would just do the work, and the note would be automatically created by an AI that was like a Jarvis style from the Avengers” (Faculty).* |
|  | *“I expect that in 10 years, I will be able to just dictate some aspects of the patient that are critical, and ChatGPT will probably create my note for me” (Faculty).* |
|  | *“If you could walk into a room and you open the note, and EPIC would say, ‘Welcome, Dr. X, this is Joe Smith. Joe Smith has been seen at X for the last 10 years, and he has these conditions’ and puts all that in, and then basically you ask Joe Smith if he has any updates he wants to add, and then it auto-populates; that would be very smart. That is truly the way you want to go, right? That is your AI software telling you a lot about the patient, and then you can list the patient's current complaint, and they just put in their current complaints while they are sitting on the iPad in the room and it is populating their chart” (Faculty).* |
| Influences from outside environment: New innovations: People focused solutions | *“I think that would be a critical piece of the training if we were to have more robust orientation or education on copy and paste for our new hires and those being trained into Epic, being able to give them a good understanding of what the right tools are to do the work that they are trying to do” (APP).* |
|  | *“Really emphasize that copy and paste is an additional resource and tool to support that work, not the primary one. Because I think a lot of new users instinctively feel that [using CP/CF is] the easiest way to do it and are not too interested or aware of additional functionalities in EPIC” (APP).* |
|  | *“If the information is readily available within the system, then I probably would not do copy/paste” (Faculty).* |
|  | *“I can write a whole note that is just dot phrase, dot phrase, dot phase, my assessment and plan, and I am done versus having to sit there and think of like, ‘Oh gosh, how do I pull that in? Do I copy and paste it? If I copy and forward it and refresh, would that make it easier?’” (Faculty).* |
|  | *“I think that is where the art of medicine comes in is to know what’s relevant and what’s not.  I think you need to copy important information that helps the next clinician” (Faculty).* |
|  | *“There is not really an intentional feedback system. We do not give them feedback on how much copy and paste they are doing, or in general, the quality of their notes, depending on how busy we are” (Faculty).* |
|  | *“I think having clear guidance and expectations and best practice workflows will help with those new faculty or new learners. I mean, if they are using the note to do a hospital summary, we should use the hospital summary section in Epic, not our note as the hospital summary.”* *(Faculty)* |
|  | *“I think that would be super valuable… I can go to those people to ask any random Epic question; I do not have to go to the Epic help desk. And honestly, it [would] eliminate the amount of overload that the Epic support team got because a lot of these challenges are pretty simple to resolve if you are somebody who is familiar with the EMR and really well versed in using it. And those members on those teams were able to guide those individuals and figure those things out without it going to the EPIC support desk and waiting in a queue” (APP).* |
| Influences from outside environment: Cultural shifts in documentation | *“[Time spent on editing notes] is just seen as an extra burden”* *(Resident/Fellow Trainee).* |
|  | *Because I have to do more things with less time.  Because no one should have to pay me to do these little clicks and checkboxes, they should pay me to spend time with the patients to enhance the patient care, to enhance the relationship, to enhance the outcomes (APP).”* |
|  | *“The problem is [note writing] becomes fatiguing if people do not do things exactly the same way every single time…. You do not have enough time to provide care and to provide the documentation. No one pays you for that. They pay you to see patients. And they should pay you to develop a relationship with patients, which can enhance outcomes. But insurance companies constrict us to what we can prove on paper. There is no quality in that” (Faculty).* |
|  | *“In addition to talking about today, if somebody has a question, if anybody has a question like, “Well, what was their hemoglobin on admit? Or where is their potassium reading?” They look at their note. They pull up their note. That is the thing that has all their information in it” (Faculty).* |
|  | *“It does not mean just because you copy/paste it stays there. It is your choice at the end when you sign the note, what you want to leave and not leave in” (Faculty).”* |
|  | *“But the key is that you read what you are copy/pasting.  Make sure you agree with what was written, and then you make sure it is in there.  Right?  So, it takes some thought to copy/pasting.  Right?  It is not just grab and put, right?  If someone has some terrible stuff in there, I do not think I want that in my notes.  I think that the responsibility for copy/pasting must be that you a) agree with what was written, and b) you think it is valuable to have in your notes.” (Faculty).* |
|  | *“And so, I would encourage policy that requires that people attribute work to the original author if they are going to utilize their texts in their own note” (Faculty).* |
|  | *“This year specifically, there has been such an increasing pressure to see more and more patients, more than any other year that I have ever been with [institution]. And that is making it harder and harder to actually document what I feel is the appropriate way to document” (APP).* |
|  | *“The only way to function really, is to be copy and pasting from yourself [and] from others” (Resident/Fellow Trainee).* |
